# Longevity

**Published:** 1880-03

**Authors:** 


### LONGEVITY.

**The** following table shows that men have attained a good old age and there is no reason to suppose that these might not be the average ages of men and women.

| | | | |
|---|---|---|---|
| Dryden, | 70 | Lewenhoeck, | 91 |
| Petrarch, | 70 | Cato, | 91 |
| Lesage, | 70 | Hans Sloane, | 93 |
| Linnæas, | 71 | Whiston, | 95 |
| Locke, | 73 | Michael Angelo, | 96 |
| La Fontaine, | 74 | Titian, | 95 |
| Rev. Dr. Wardlow, | 75 | Isocrates, | 98 |
| Handel, | 75 | Elisha, | 100 |
| Reaumer, | 75 | Hervelias, | 100 |
| Gallileo, | 78 | Fontenelle, | 100 |
| Swift, | 78 | Zeno, | 100 |
| Roger Bacon, | 78 | Terentia, | 103 |
| Corneille, | 78 | Stender, | 103 |
| Marmontel, | 79 | Helen Gray, | 105 |
| Solon, | 80 | Georgias, | 107 |
| Thucydides, | 80 | Thomas Garrick, | 108 |
| Anacreon, | 80 | Democritus, | 109 |
| Juvenal, | 80 | Joseph, | 110 |
| Kant, | 80 | Joshua, | 110 |
| Pindar, | 80 | A. Serush, | 111 |
| Young, | 80 | Mittelstedt, | 112 |
| Willard, | 80 | H. Thauper, | 112 |
| Sophocles, | 80 | R. Glen, | 115 |
| Plato, | 81 | Moses, | 120 |
| Buffon, | 81 | Prastus, King of Poland, | 120 |
| Goethe, | 82 | Sarah, | 127 |
| Dr. Chas. Caldwell, | 82 | Ishmael, | 137 |
| Claude, | 82 | Effingham, | 144 |
| West, | 82 | Countess of Desmond, | 145 |
| Franklin, | 84 | Drakenberg, | 146 |
| Metastasio, | 84 | Jacob, | 147 |
| Herschell, | 84 | Thomas Parr, | 153 |
| Anacreon, | 85 | Thomas Damme, | 154 |
| Newton, | 85 | Epimenides, | 157 |
| Voltaire, | 85 | Henry Jenkins, | 169 |
| Halley, | 86 | John Rovin, | 172 |
| Simeon, | 90 | Abraham, | 175 |
| Fabius, | 90 | Isaac, | 180 |
| Eli, | 90 | Peter Torten, | 185 |
| Protagoras, | 90 | Monga of Kentigen, | 185 |
| Livia, | 90 | | |

